# Technology and Method Optimization for Foot–Ground Contact Force Detection in Wheel-Legged Robots

**DOI:** 10.3390/s25134026

**Published:** 2025-06-27

**Authors:** Chao Huang, Meng Hong, Yaodong Wang, Hui Chai, Zhuo Hu, Zheng Xiao, Sijia Guan, Min Guo

**Affiliations:** 1 Hubei Provincial Engineering Research Center of Robotics & Intelligent Manufacturing, Wuhan University of Technology, Wuhan 430070, China; 297179@whut.edu.cn (C.H.); hongmeng@whut.edu.cn (M.H.); 332699@whut.edu.cn (Y.W.); reallylaugh@whut.edu.cn (Z.X.); 2 School of Control Science and Engineering, Shandong University, Jinan 250061, China; chaimax@sdu.edu.cn; 3 China Aerospace Science and Industry Corporation Second Research Institute, Beijing 100039, China; guansijia@azspace.cn

**Keywords:** quadruped robot, contact force estimation, piezoelectric film sensors, neural network, Gaussian process regression, artificial neural network

## Abstract

Wheel-legged robots combine the advantages of both wheeled robots and traditional quadruped robots, enhancing terrain adaptability but posing higher demands on the perception of foot–ground contact forces. However, existing approaches still suffer from limited accuracy in estimating contact positions and three-dimensional contact forces when dealing with flexible tire–ground interactions. To address this challenge, this study proposes a foot–ground contact state detection technique and optimization method based on multi-sensor fusion and intelligent modeling for wheel-legged robots. First, finite element analysis (FEA) is used to simulate strain distribution under various contact conditions. Combined with global sensitivity analysis (GSA), the optimal placement of PVDF sensors is determined and experimentally validated. Subsequently, under dynamic gait conditions, data collected from the PVDF sensor array are used to predict three-dimensional contact forces through Gaussian process regression (GPR) and artificial neural network (ANN) models. A custom experimental platform is developed to replicate variable gait frequencies and collect dynamic contact data for validation. The results demonstrate that both GPR and ANN models achieve high accuracy in predicting dynamic 3D contact forces, with normalized root mean square error (NRMSE) as low as 8.04%. The models exhibit reliable repeatability and generalization to novel inputs, providing robust technical support for stable contact perception and motion decision-making in complex environments.

## 1. Introduction

Quadruped robots are widely used in applications such as factory inspection, emergency rescue, and warehouse transportation due to their excellent locomotion capabilities [1]. Most current quadruped robots adopt legged structures. On rough terrains, their superior off-road capability and terrain adaptability significantly enhance mobility [2]. However, such mechanisms suffer from low energy efficiency and complex gait control when operating on flat surfaces. In contrast, wheeled robots, though lacking flexibility in unstructured environments, offer higher speed and better energy efficiency compared to legged robots [3]. Wheel-legged robots combine the advantages of both: using wheels for fast locomotion on relatively flat ground, and switching to legged motion to overcome obstacles such as steps and pits. They are typically composed of actuated wheels, extendable legs, sensor systems, and dual-mode control architectures, which enable flexible transitions between wheeled and legged locomotion based on varying environmental conditions [4]. This enables autonomous transitions between locomotion modes under varying terrain conditions [5,6]. Wheel-legged robots are equipped with dual independent control systems and combined wheel–leg structures. However, they still face several core technical challenges, particularly in achieving smooth and robust transitions between locomotion modes. One major difficulty lies in the accurate modeling of wheel–leg–terrain interaction, including ground contact forces, dynamic friction estimation, and foot-end impact control. These factors are vital not only for maintaining balance and minimizing slip but also for enabling predictive and adaptive motion planning [7]. Recent advances have addressed some of these issues. For example, Chen et al. [8] proposed a sliding mode control framework for foot-end trajectory consensus under variable topological constraints. Wei et al. [9] developed a Kalman-filter-based adaptive contact force estimator for robotic systems with dynamic model imperfections, with potential extensions to mobile hybrid robots. Liu et al. [10] introduced a robust model predictive control (RMPC) framework for wheel-legged hybrid vehicles, incorporating frictional uncertainties and contact force disturbances. Liu and Zhang [7] focused on impact dynamics modeling for hybrid motion planning. Notably, a growing body of literature has modeled foot–ground interaction more accurately using dissipative and nonlinear contact models, originally developed for bipedal systems but increasingly applicable to wheel-legged robots. Corral et al. [11] studied passive biped walking dynamics by comparing various contact and friction models, identifying the dissipative nonlinear Flores model and Bengisu friction law as best suited for realistic, smooth contact modeling. These insights are valuable for improving foot-end impact control in hybrid robots. Additionally, Moreno [12] discusses a multibody modeling approach for systems with multiple, simultaneous contacts and impacts, such as those in billiard-ball-like interactions. The authors evaluate smooth contact force models, including those that account for friction and energy dissipation, offering further insights into selecting efficient and physically plausible impact models. This modeling framework can inform wheel-legged robot design, particularly in handling complex terrain interactions with multiple points of contact. Abad et al. [13] proposed a quasi-static model and trajectory optimization method that allows UGVs to optimize the configuration angles of their wheel-legs under different terrain conditions, thereby achieving a minimum-torque strategy to save energy. They also analyzed the relationship between ground–wheel contact friction and normal forces. These studies emphasize the importance of contact intelligence, compliant control, and integrated sensing. In recent years, integrating multi-sensor perception and control strategies has emerged as a critical approach to improving robotic adaptability and stability on complex terrains. For instance, Tan et al. [14] proposed a locomotion framework that combines visual and proprioceptive feedback, using a single forward-facing camera to generate a heightmap while regulating gait frequency and speed through proprioceptive cues, enabling stable and natural transitions across uneven terrain. In contrast, Wang et al. [15] demonstrated that terrain attitude estimation and contact state detection can be effectively achieved using only proprioceptive inputs—such as joint torque, IMU, and kinematics—without relying on any visual sensors, enhancing the robustness of quadruped robots under challenging ground conditions. These findings collectively underscore that accurate foot-end contact modeling and intelligent control are fundamental to improving locomotion stability, energy efficiency, and terrain adaptability in wheel-legged robots.

In previous studies on foot-end perception of quadruped robots, researchers have largely relied on external sensors such as vision cameras, LiDAR, inertial measurement units (IMUs), and joint encoders to enhance environmental adaptability and decision-making capabilities [16]. However, wheel-legged robots often operate in highly unstructured or visually degraded environments, where these external sensors are prone to occlusion, lighting issues, or signal loss [17]. In such scenarios, contact measurement provides a robust and direct perception mechanism by capturing the physical interaction between the robot and terrain in real time. Notably, recent studies have highlighted the benefits of contact force sensing in locomotion optimization. For instance, Pepe et al. [18] proposed a genetic-algorithm-based method that directly optimizes the ground reaction forces of quadruped robots to identify energetically efficient gaits. Their results demonstrate that knowledge of contact forces can not only improve energy efficiency but also facilitate the emergence of biologically plausible gaits such as walking and trotting under varying speed conditions. Integrating such force-based strategies into wheel-legged robots offers promising avenues for enhancing motion adaptability, especially when traditional exteroceptive sensing becomes unreliable. Therefore, early studies tended to estimate the contact state between the robot and the environment indirectly by combining proprioceptive sensors (such as joint angular velocity and center-of-mass velocity) with various filtering algorithms (e.g., Kalman filters) [19]. However, since proprioceptive sensors cannot directly perceive external environmental information, the accuracy of such methods is limited under extreme conditions. In recent years, contact-based sensors specifically designed for quadruped robots have gradually attracted attention. Owing to the distinctive mechanical design of wheel-legged robots, their foot-end typically comprises a continuously rotating tire rather than a rigid, flat-foot structure as employed in conventional legged robots. This tire-based configuration exhibits substantial deformation and complex ground-contact dynamics under varying load conditions, rendering it unsuitable to be modeled as a simple rigid body in force analysis. Furthermore, the curved geometry and rotational motion of the tire pose considerable challenges for the direct integration of conventional foot-end sensors—such as force, pressure, or tactile sensors—commonly adopted in quadruped robotic systems. Consequently, wheel-legged platforms necessitate the development of specialized sensing methodologies capable of accommodating the unique kinematic and structural characteristics of their foot-end components [20,21]. To address this challenge, recent research has focused on leveraging smart tire technologies, which utilize sensors integrated into the tire to measure deformation or strain, thereby enabling more accurate estimation of tire–ground contact forces [22]. Currently, smart tire systems commonly employ triaxial accelerometers [23], optical sensors [24], strain gauges [25], and polyvinylidene fluoride (PVDF) piezoelectric film sensors [26]. Among these, PVDF sensors have been widely adopted in tire applications due to their flexibility, low cost, and high sensitivity. Mechanical deformation applied to the PVDF film generates a voltage difference between its surfaces. Yi et al. [26] proposed a PVDF sensor for measuring stress on the inner liner of a tire, with the stress measurements interpreted using a friction force model. Their results demonstrated the feasibility of PVDF-based tread deformation sensing. Similar PVDF-based tire sensor systems have also been reported in the studies by Armstrong [27] and Toplar [28].

In estimating the tire state at the foot-end of wheel-legged robots using smart sensing technologies, a core challenge lies in establishing an effective mapping between sensor signals and tire states. The three-dimensional contact force and contact position are key parameters for contact state estimation [29]. If such information can be directly measured or indirectly inferred through smart tire technologies, it would significantly reduce reliance on external sensors, simplify the state estimation process, and enhance both accuracy and system robustness [30]. However, tires are highly complex nonlinear systems characterized by material, contact, and geometric nonlinearities, making them difficult to model accurately using traditional mathematical approaches [31]. Model-based methods rely on physical models to characterize tire–road interactions. For instance, Hong et al. [32] proposed a brush-model-based algorithm to estimate the tire–road friction coefficient by relating lateral deflection to lateral force and aligning torque. Although such models offer physical interpretability, they often require simplified formulations and impose high computational demands for real-time applications. Feature-based methods extract key indicators from sensor measurements and use prior knowledge for state identification, making them the most widely used estimation approach []. For example, Niskanen and Tuononen [33] identified different phases of the tire contact patch using three characteristic peaks in acceleration signals. Morinaga et al. [34] estimated lateral force based on the ratio of contact lengths. However, these methods are highly sensitive to environmental factors such as temperature, tire pressure, and wear, requiring frequent recalibration of thresholds and filter parameters, which limits their robustness. In contrast, machine learning approaches are particularly well-suited for handling complex nonlinear problems. To overcome the modeling challenges posed by nonlinear tire behavior, neural network models such as multilayer perceptrons (MLPs) or convolutional neural networks (CNNs) can be employed. These models take raw sensor data—such as acceleration and strain signals—as input, and output key contact parameters like three-dimensional contact force or foot-end position, enabling more robust and accurate estimation without relying on manual feature extraction [35,36]. Gaussian process regression (GPR), a non-parametric method based on Bayesian theory, is capable of modeling high-dimensional inputs and nonlinear responses while simultaneously providing uncertainty estimates. As a result, GPR shows great promise in tire state estimation and its downstream applications, such as model predictive control (MPC) [37].

In previous research, Wang [20] designed a heuristic contact position estimator for wheel-legged robots by analyzing the deformation characteristics of the foot-end under loading through finite element analysis and employing an array of strain sensors to detect both the contact position and the normal force, thereby validating the feasibility of the approach. However, this method did not address the estimation of tangential forces, the adaptability to different tire types, nor the systematic influence of sensor placement on estimation performance. In fact, the choice of sensor placement is critical to the smart tire’s ability to capture dynamic signal features. Strategically positioning sensors in areas with higher responsiveness can significantly enhance the accuracy and robustness of foot-end state estimation. Therefore, identifying the optimal mounting positions for PVDF sensors holds substantial research and application value [38]. Global sensitivity analysis (GSA) is an effective tool for quantifying the influence of input uncertainty on output responses and is widely used in engineering design optimization [39]. Among various GSA techniques, Sobol’s variance-based sensitivity analysis method [40] is the most commonly applied. However, its high computational cost has limited its practical application in engineering. To address this, Zhao [41] proposed a method based on multiplicative dimension reduction and Gaussian quadrature grids, which significantly reduces computational demand. This study adopts Zhao’s approach to perform global sensitivity analysis on the sensor placement for wheel-legged robot foot-ends, aiming to determine the optimal configuration [41].

Building upon the contributions of previous research in both UGV and wheel-legged robot domains, and recognizing the limitations that remain—particularly in accurate terrain contact modeling and real-time adaptability—we aim to further enhance the adaptability of wheel-legged robots by optimizing the application of PVDF strain sensors for foot-end contact detection. This paper is structured as follows: Section 2 introduces the finite element simulation modeling process of smart tires and applies global sensitivity analysis to determine optimal sensor placement; Section 3 presents the experimental platform setup used to verify the reliability of contact position estimation; Section 4 describes physical testing under varying gait frequencies to assess the robustness of existing models in complex environments; the conclusions are provided in Section 5.

## 2. Foot-End Modeling and Validation for Wheel-Legged Robots

### 2.1. Finite Element Modeling of Smart Tires and Strain Distribution Analysis

**Figure 1:**
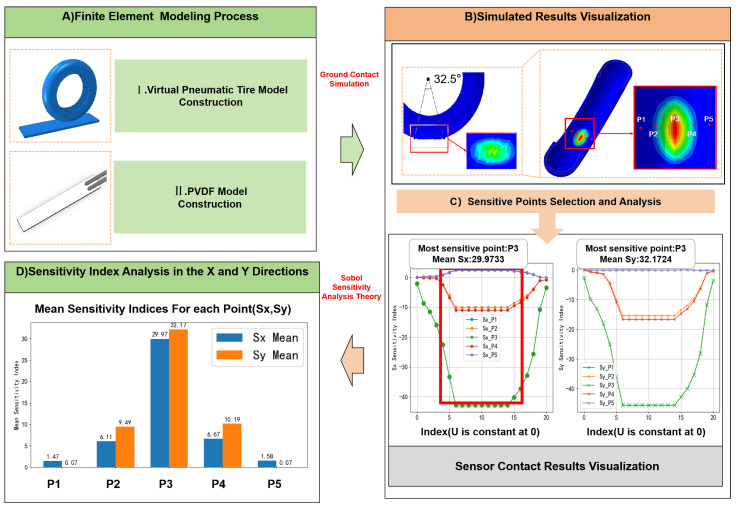
Finite element analysis process of the wheel-legged foot-end. (**A**) Finite element modeling of the tire–ground contact process. (**B**) Visualization of simulation results with reference points P1–P5 selected based on strain magnitude. (**C**) Simulated sensor stress results at different contact positions; as highlighted in the red box, P3 exhibits the most prominent strain response in both x- and y-directions. (**D**) Sensitivity indices of strain with respect to contact depth in the x- and y-directions at each selected location, with P3 reaching 29.97 and 32.17.

To qualitatively analyze the deformation of the tire surface along the circumference during ground contact in wheel-legged robots, finite element modeling was conducted using ABAQUS, as illustrated in Figure 1. In the simulation setup, a rigid analytical body was placed beneath the tire and assigned a constant velocity to compress the tire mesh by a specified amount. During the analysis, linear elastic material models were deemed inadequate for accurately capturing the nonlinear elastic behavior of rubber. Therefore, the tire material was modeled as an isotropic hyperelastic material, with parameters detailed in Table 1. The Mooney–Rivlin constitutive model was selected due to its balance between simplicity and accuracy, enabling effective representation of the nonlinear stress–strain response under deformation with relatively few material parameters [42,43]. The model parameters used in the FEA simulation are summarized in Table 1. The International Rubber Hardness Degree (IRHD) value of 43 reflects the material’s stiffness and is commonly used in elastomer characterization. The constants $C_{10}$ = 0.144 MPa and $C_{01}$ = 0.036 MPa define the strain energy function based on the first and second strain invariants. The parameter D1 = 0 corresponds to the bulk modulus and represents the assumption of material incompressibility, which simplifies computation without compromising model fidelity [44].

The strain energy function is given by:

(1) $$W=C_{10}(I_{1}-3)+C_{01}(I_{2}-3)$$

Here, $C_{10}$ and $C_{01}$ are material constants, representing the first and second invariants of the deformation tensor, respectively.

**Figure 2:**
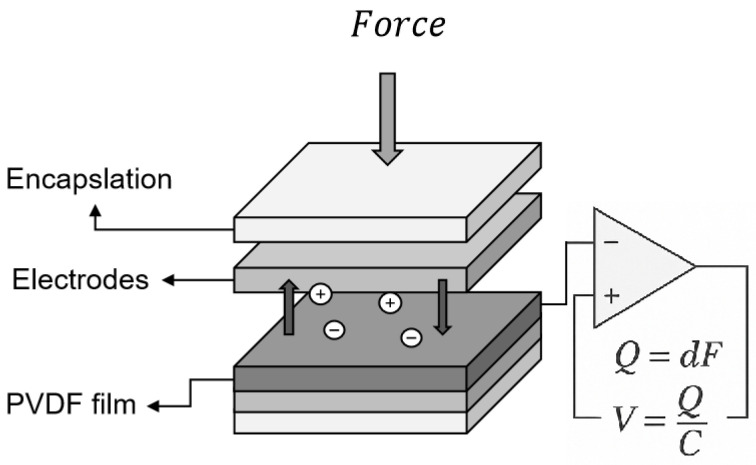
PVDF sensor structure.

As illustrated in Figure 2, the PVDF sensor typically consists of a five-layer structure, including upper and lower plastic protective layers, upper and lower electrode layers, and a central PVDF piezoelectric film. The protective layers serve to shield the film from mechanical damage during operation, while the electrode layers are used to extract electrical signals generated by the piezoelectric effect. Since the protective and electrode layers have minimal influence on the piezoelectric response itself, the modeling process in this study simplifies the sensor structure by modeling only the central PVDF film. The schematic of the simplified PVDF model used in this study is shown in the upper-left corner of Figure 1.

The finite element visualization shown in Figure 1 presents the simulation results of the foot-end tire under an internal pressure of 0.2 MPa. It can be observed that when the outer surface of the tire undergoes deformation, the equivalent stress distribution on the inner surface at the contact region exhibits a distinct Gaussian profile. In this study, the most representative circumferential position, 32.5°, is selected for illustration. Within the strain-affected region, five sensor installation points (P1–P5) are designated to analyze the strain behavior around the deformation center. Additionally, two more points—symmetrically and asymmetrically located with equal angular offsets—are selected for comparative evaluation. The results show that the strain difference between these paired points increases as the contact location deviates from the central position.

### 2.2. Global Sensitivity Analysis for Optimal Sensor Placement

In Figure 1B, the strain distribution during tire–ground contact is shown. Based on the varying levels of strain, analysis points P1 to P5 are selected sequentially from the center toward the sides. Global sensitivity analysis (GSA) is employed in this study, with the global sensitivity index (GSI) used as the metric to quantify the influence of sensor placement positions. The total variance V(Y) of the model output Y can be decomposed into contributions from each input variable Xi and their interaction terms. In the context of evaluating the finite element model of the tire, the input variables correspond to the contact depths, while the output variables represent the strain at specific internal locations within the tire, denoted as Sx or Sy. The sensitivity index Si quantifies the proportion of strain variation attributable to changes in a specific contact depth. Accordingly, the output variances of Sx and Sy can be expressed as:

(2) $$S_{x}=f(P_{1},P_{2},P_{3},P_{4},P_{5})$$

(3) $$S_{y}=f(P_{1},P_{2},P_{3},P_{4},P_{5})$$

(4) $$V(S_{x})=\sum_{i=1}^{5}V_{1}+\sum_{i<j}^{n}V_{ij}+\sum_{i<j<k}^{n}V_{ijk}+\ldots +V_{1,2,3,4,5}$$

(5) $$V(S_{y})=\sum_{i=1}^{5}V_{1}+\sum_{i<j}^{n}V_{ij}+\sum_{i<j<k}^{n}V_{ijk}+\ldots +V_{1,2,3,4,5}$$

Here, Vi represents the portion of the variance in the output strain Sx that is solely attributable to the input variable, specifically the contact depth at a given location. The term Vij refers to the interaction effect between the input variables Pi and Pj. The total variance of the output strain in the x-direction is denoted as V(Sx), while V(Sy) similarly represents the total variance of the output strain in the y-direction.

The first-order sensitivity index $S_{i}$ represents the contribution of a single input variable $X_{i}$ to the total variance of the output Y. It is suitable for analyzing the relationship between contact depth and strain at a single sensor location, and for evaluating the relative influence of that point. It is defined as:

(6) $$S_{i}=\frac{V_{i}}{V(Y)}=\frac{V_{X_{i}}(E_{X_{\sim i}}(Y|X_{i}))}{V(Y)}$$

Here, $E_{X_{\sim i}}$ denotes the expectation over all other variables except $X_{i}$. The corresponding visualization and experimental data are shown in the lower-left of Figure 1 and in Table 2.

The analysis results indicate that point P3 exhibits the highest average sensitivity indices in both the x- and y-directions (29.9733 and 32.1724, respectively), suggesting that this location is the most responsive to changes in sensor signals. In contrast, the average sensitivity indices for the other locations (P1, P2, P4, and P5) are comparatively lower in both directions. The sensitivity analysis results illustrated in Figure 1C further confirm the effectiveness of P3 as a highly sensitive point for detecting strain variations inside the tire.

**Figure 3:**
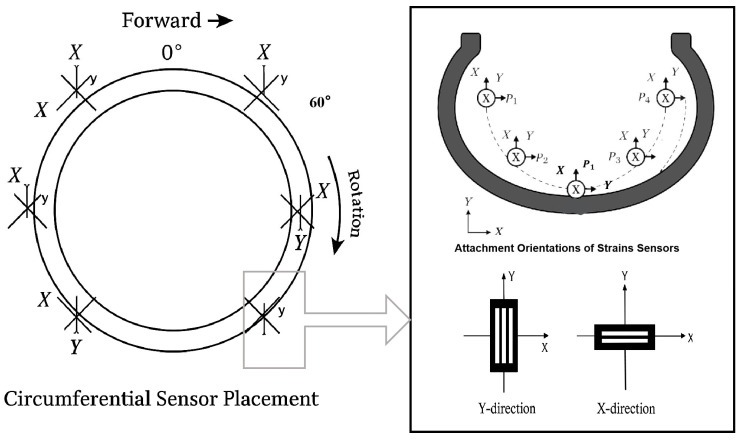
Sensor placement.

Based on the above analysis of sensor placement, this study adopts the configuration shown in Figure 3, where sensor units are arranged every 60° around the tire circumference. Each unit consists of two sensors oriented tangentially and circumferentially to capture strain in both the x- and y-directions during rolling and stepping motions at the foot end.

## 3. Experimental Validation of Sensor Placement and Contact Response

### 3.1. Sensor Placement Analysis and Validation

Based on the analysis results from the previous chapter, this section conducts experimental validation of sensor placement reliability on the foot-end of the wheel-legged robot under real-world conditions. Specifically, it compares the voltage output trends of different sensor groups under varying independent variables such as tire pressure and external load. For any given location on the tire, the strain–voltage relationship for sensors arranged in the tangential and circumferential directions can be described as:

(7) $${\;V}_{PVDF}=f(A1,A2)$$

(8) $$V_{PVDF}^{\prime }=f({A1}^{\prime },{A2}^{\prime })$$

Here, $V_{PVDF}$ and $V_{PVDF}^{\prime }$ represent the output voltages under tangential and circumferential sensor arrangements, respectively, while A1 and A2 denote the independent variables such as tire pressure and load.

**Figure 4:**
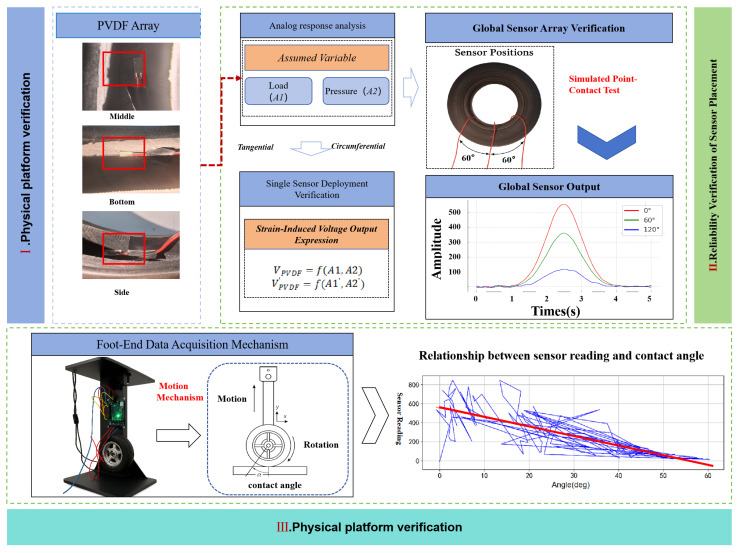
Sensor placement verification process. (**I**) Physical drawing of PVDF arrangement in pneumatic tire, with red frames used to highlight the sensor positions within the tire. (**II**) The output of strain gauge at different embedded positions. (**III**) The left figure shows the test platform and schematic diagram, and the right figure shows the contact position dataset distribution. The red lines represent the general trend between the collected data and the contact angle.

**Figure 5:**
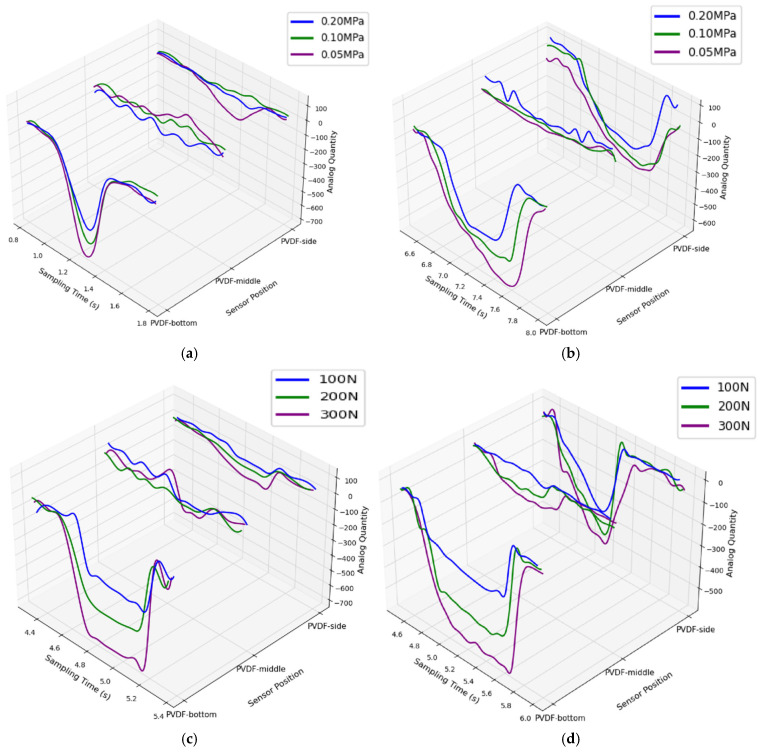
Reliability verification of single-point sensor placement. (**a**) Circumferential arrangement (tire pressure). (**b**) Tangential arrangement (tire pressure). (**c**) Circumferential arrangement (load). (**d**) Tangential arrangement (load).

As shown in the first part of Figure 4, considering the structural symmetry of the tire, this study primarily evaluates the reliability of PVDF sensors arranged on a single side of the inner tire—specifically at the crown, shoulder, and sidewall regions. In Figure 5, the voltage outputs from various sensor configurations positioned at the same circumferential location are validated under controlled independent variables. Under the given test conditions, as the external load increases, the voltage responses of all sensor positions show a general upward trend. This phenomenon is mainly attributed to the increased load causing more pronounced local deformation in the tire, thereby generating higher voltage outputs. Conversely, as the tire pressure increases, the overall stiffness of the tire also increases, which suppresses local deformation under load and impact, leading to reduced strain response and thus lower voltage output from the sensors. Overall, sensors arranged in both the tangential and circumferential directions effectively reflect stress variations at individual foot-end contact points. Furthermore, as shown in the upper-right of Figure 4, the sensors arranged at 60° intervals along the tire circumference produce output results that generally meet the perception requirements of the wheel-legged robot foot-end during contact events.

### 3.2. Experimental Platform and Reliability Validation

As shown in the third section of Figure 4, a custom experimental platform is introduced in this study to simulate the dynamic foot–ground contact behavior of wheel-legged robots under varying gait frequencies. The test rig is designed to apply controlled forces in specified directions, ensuring that the tire follows a predefined trajectory while maintaining a consistent loading force, thereby enabling controlled and repeatable dynamic contact experiments. The platform consists of a flat contact surface placed horizontally, a motor-driven reciprocating push-end, pressure sensors mounted on the surface, and a rotation sensor (encoder) installed on the push-end. The tire is fixed to the reciprocating push-end, which cyclically presses and lifts it from the ground. During this process, pressure data is collected by sensors embedded in the ground plate, while the encoder measures the relative rotation angle between the tire and the push-end. The reciprocating motion is generated via a crank–cam linkage driven by a servo motor through a speed reducer, simulating vertical leg movement. Each full rotation of the cam triggers one contact cycle of the robot foot with the ground, producing continuous data that reflects real-time contact changes. By adjusting motor speed and tire deformation, different ground impact scenarios can be simulated. In addition, to analyze the relationship between strain and contact force at different positions in later stages, a PVDF sensor embedded inside the tire and a three-dimensional force sensor fixed on the contact surface were used during experiments. These sensors provided more comprehensive and high-resolution data for describing the contact force distribution.

The experiment focuses on the shear direction (*x*-axis), with the tire rotating in fixed angular increments. Sensor data are collected from approximately 1/12 of the tire’s surface contact region. At each contact position, the sensor records data under identical load conditions in a direction perpendicular to the plane of the tri-axial force sensor. This setup allows for linear vertical translation and axial rotation of the tire, ensuring consistent and controllable data acquisition at various contact angles. The range of contact positions and normal reaction forces used in this study is summarized in Table 3.

**Figure 6:**
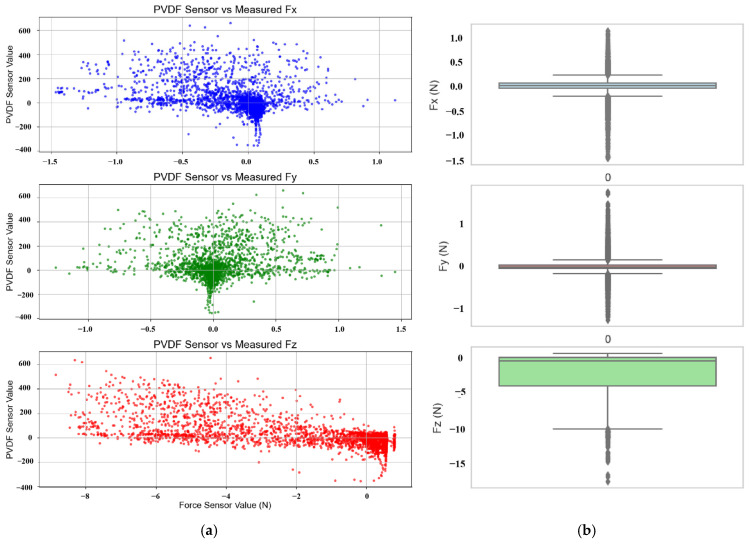
Comparative distribution of contact force datasets. (**a**) Distribution of 3D contact force dataset. (**b**) Box plots of contact force components.

A comparative analysis was conducted on the collected dynamic contact data. As shown in Figure 4, under high-frequency gait conditions, sensor readings exhibit a relatively linear relationship with contact position. However, Figure 6a reveals that the relationship between sensor readings and three-dimensional contact forces is highly nonlinear, making it difficult to predict using simple fitting methods. Figure 6b presents box plots of the three-axis contact forces under low-frequency conditions. The data distributions of $F_{x}$ and $F_{y}$ are relatively stable, with fewer outliers and narrower ranges, particularly for $F_{x}$, indicating lower variability. In contrast, $F_{z}$ shows a more dispersed distribution, with prominent outliers in the negative value region, reflecting large fluctuations in the normal force likely caused by multiple influencing factors. Therefore, special attention should be given to the treatment of outliers in the $F_{z}$ variable during subsequent modeling.

## 4. Estimation and Validation of Foot-End Contact Characteristics

### 4.1. Contact Feature Data Processing

Based on the dataset collected in the previous chapter, preprocessing of the sensor signals is required before feeding them into the model for training. This step ensures that the data quality meets the requirements for model training and helps improve prediction accuracy and convergence speed. Under highly dynamic and unstable data acquisition conditions, calibration is performed by setting the initial reading of each sensor as a reference baseline. Specifically, given an initial reference value $v_{\mathrm{init}}$, each subsequent measurement $v_{\mathrm{measured}}(t)$ is calibrated using the following equation:

(9) $$v_{\mathrm{calibrated}}\left(t\right)=\left(v_{\mathrm{measured}}\left(t\right)-v_{\mathrm{init}}\right)\times \left(-1\right)$$

Here, $v_{\mathrm{calibrated}}(t)$ represents the calibrated output value, and $v_{\mathrm{measured}}(t)$ denotes the original measurement. The multiplication by −1 serves as a sign inversion to adjust the sensor’s response direction, ensuring that the measured value accurately reflects the physical quantity and eliminates initial bias. To further smooth the raw data x(t), a moving average filter is applied as follows:

(10) $$\hat{x}\left(t\right)=\frac{1}{N}\sum_{i=0}^{N-1}\;x\left(t-i\right)$$

Here, $\hat{x}(t)$ represents the smoothed data, N is the window size, and x(t) is the raw data. By replacing the current value with the average of N surrounding points, high-frequency noise can be effectively filtered out, thereby enhancing data smoothness. In addition, the z-score method is used to detect outliers. The corresponding formula is given as follows:

(11) $$z_{i}=\frac{x_{i}-\mu }{\sigma }$$

**Figure 7:**
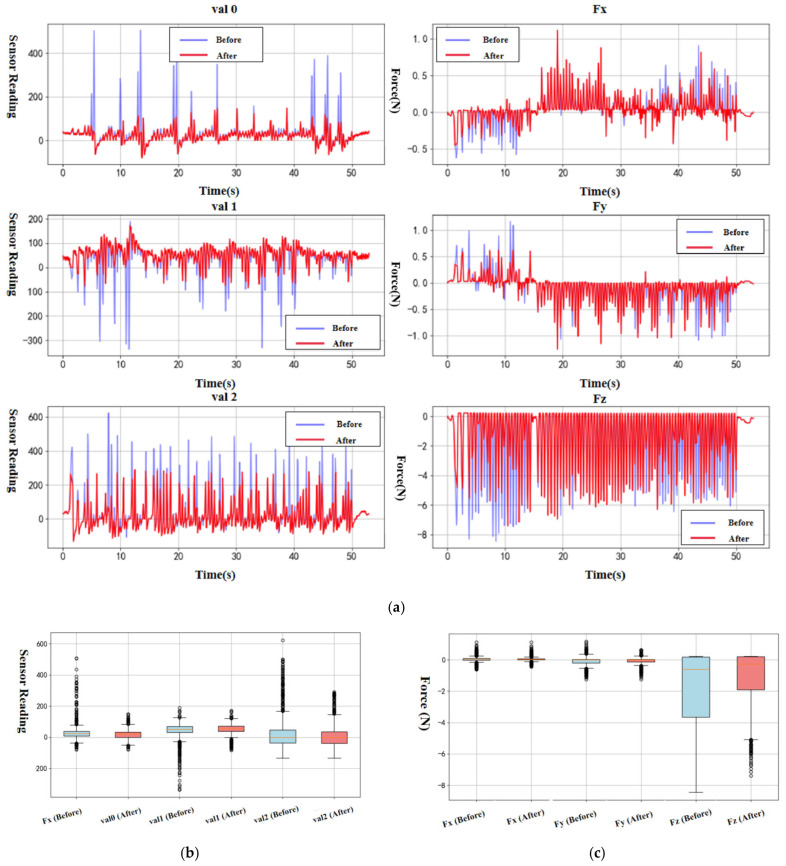
Results after noise filtering and outlier removal from dynamic data. (**a**) Performance of preprocessed data on test dataset. (**b**) Comparison of sensor data before and after preprocessing (box plot). (**c**) Comparison of measured force data before and after preprocessing (box plot).

Here, $x_{i}$ denotes a data point, μ is the mean, and σ is the standard deviation. If $|z_{i}|>k$ (where k is a predefined threshold), the corresponding value $x_{i}$ is considered an outlier and removed to prevent interference with subsequent model training. The results are shown in Figure 7. As illustrated by the figure and the box plot, the processed dataset is noticeably smoother, and the number of outliers is significantly reduced.

Subsequently, the dataset $\left\{x_{1},x_{2},\ldots ,x_{n}\right\}$ is standardized and normalized to ensure consistency in the scale of all features. The value of ${\hat{x}}_{i}$ is computed as follows:

(12) $${\hat{x}}_{i}=\frac{x_{i}-\mu }{\sigma }$$

(13) $${\hat{x}}_{i}=\frac{x_{i}-x_{\min}}{x_{\max}-x_{\min}}$$

Here, μ represents the mean,σ is the standard deviation, and $x_{\min}$ and $x_{\max}$ denote the minimum and maximum values of the dataset, respectively.

Due to inherent sampling frequency differences between the flexible sensors and the ground-truth force sensors, there are often inconsistencies in timestamps during actual data acquisition. Therefore, a linear interpolation method is employed to align all sensor data to a unified time axis. The original data $x_{i}(t)$ are mapped onto a time grid $t_{1},t_{2},\ldots ,t_{m}$, and the interpolation formula is given as follows:

(14) $${\hat{x}}_{i}(t_{k})=x_{i}(t_{j})+\frac{\left(t_{k}-t_{j}\right)}{\left(t_{j+1}-t_{j}\right)}\left(x_{i}(t_{j+1})-x_{i}(t_{j})\right)$$

Here, $t_{k}$ denotes the interpolation time point, $t_{j}$ and $t_{j+1}$ are the adjacent measured time points, and $x_{i}(t_{j})$ and $x_{i}(t_{j+1})$ are the corresponding sensor data values at those times. This step produces a time-aligned dataset, facilitating subsequent analysis and modeling.

### 4.2. Dynamic Contact Force Estimation

In the time-series prediction task, to enhance the model’s learning capability, this study introduces three significant features based on sliding window and lag analysis: slope features and rolling statistical estimates (including rolling mean and rolling variance). The corresponding formulas are as follows:

(15) $${\mathrm{slope}}_{val_{i}}(t)=\left|\frac{\Delta {\mathrm{val}}_{i}}{\Delta t}\right|=\left|\frac{{\mathrm{val}}_{i}(t)-{\mathrm{val}}_{i}(t-1)}{t-(t-1)}\right|$$

(16) $${\mathrm{mean}}_{val_{i}}(t)=\frac{1}{w}\sum_{k=t-w+1}^{t}\;{\mathrm{val}}_{i}(k),w=10$$

(17) $${\mathrm{var}}_{val_{i}}(t)=\frac{1}{w-1}\sum_{k=t-w+1}^{t}\;{\left({\mathrm{val}}_{i}(k)-{\mathrm{mean}}_{val_{i}}(t)\right)}^{2},w=0$$

Here, i=0,1,2 denotes the input variable. The slope feature is computed by calculating the difference between consecutive values, with NaN inserted at the first position to maintain consistency in sequence length. Rolling statistical features (mean and variance) are computed using a sliding window (with a default window size of 10), enabling the capture of local statistical properties through the rolling mean and rolling variance.

**Figure 8:**
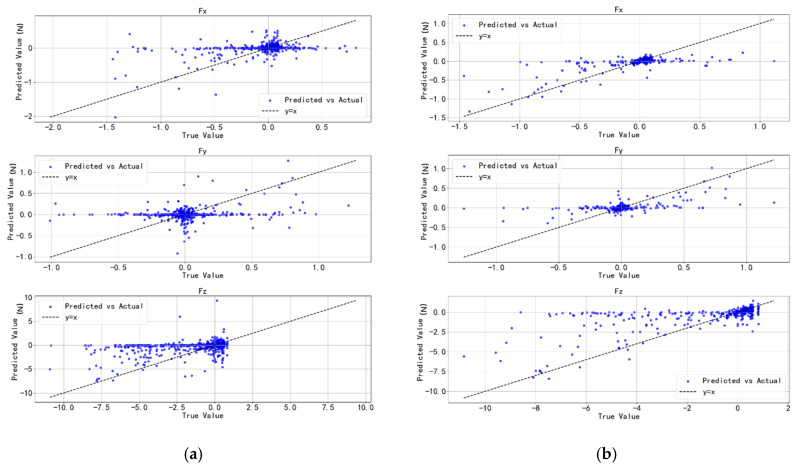
Optimization process of the GPR estimation model. (**a**) Estimation results of GPR on the test set. (**b**) Performance of the GPR model with multi-feature enhancement on the test set.

As shown in Figure 8, prior to training the GPR model, the data were formatted by contact angle and filtered to retain only six-dimensional force signals, with non-contact data below a specified threshold removed. Hyperparameters were manually tuned using a validation set, with the final values set to $\sigma_{n}$ = 0.1, $\sigma_{f}=1.0$, and l = 1.0. The model was trained over 200 iterations on the training set, using one out of every ten data points for validation, and an additional 20% of the data was reserved for testing. To enhance the model’s ability to predict multi-feature time-series data, a systematic approach was adopted to optimize the GPR hyperparameters. The model maximized the log marginal likelihood and employed the L-BFGS-B algorithm to search for the optimal length scale and noise level of the RBF kernel within a predefined range. To avoid convergence to local optima, a random restart strategy was introduced with 50 restarts, improving both robustness and global search capability. All initial values and boundary conditions were selected based on data characteristics, balancing model flexibility and computational efficiency.

To evaluate the impact of feature expansion on model performance, an ablation study was conducted, comparing four types of input configurations: raw sensor data, data without slope features, data without rolling statistical features, and data with all feature combinations. The normalized root mean square error (NRMSE) was used as the evaluation metric. As shown in Table 4, the results indicate that a richer feature set leads to significantly improved estimation accuracy, with notable reductions in NRSME across all three force components—Fx, Fy, and Fz.

**Figure 9:**
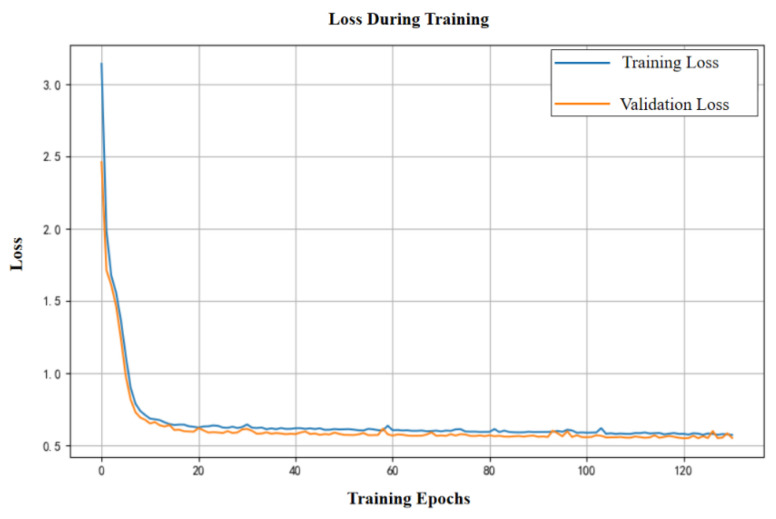
Training loss curve of the ANN model.

To improve the performance of sensor signal estimation, a comparative model based on an artificial neural network (ANN) was introduced. A systematic evaluation was conducted across different combinations of activation functions, number of nodes, and network layers. Candidate architectures with over 90% accuracy were first identified using a small-scale dataset (2131 samples), followed by training and validation on the full dataset (6549 samples). As shown in Figure 9, the model converged rapidly within the first 20 epochs, with training and validation losses decreasing synchronously, indicating good fitting performance. Although slight overfitting was observed (with validation loss around 0.1–0.2), the training process remained stable and effectively captured feature patterns.

Several trends were observed during iterative experiments with different ANN designs. In terms of node and layer configuration, architectures that employed a “first increasing then decreasing” or a “monotonically varying” number of nodes from the input to the output layer generally achieved higher prediction accuracy. Regarding network size and performance, large-scale ANNs—with five or more layers and at least 25 nodes per layer—typically exhibited lower accuracy, longer training times, and a higher tendency toward overfitting, resulting in overall performance inferior to smaller networks. With respect to activation functions, the choice among sigmoid, ReLU, ELU, and softmax had minimal impact on model accuracy for hidden layers. However, the activation function of the output layer needed to be selected according to the range and normalization of the target data. Regarding loss functions, both mean squared error (MSE) and Poisson negative log-likelihood loss (PoissonNLLLoss) were evaluated. The MSE loss function consistently delivered slightly higher prediction accuracy—typically a few percentage points better—compared to the Poisson loss function under the same data and ANN structure.

**Figure 10:**
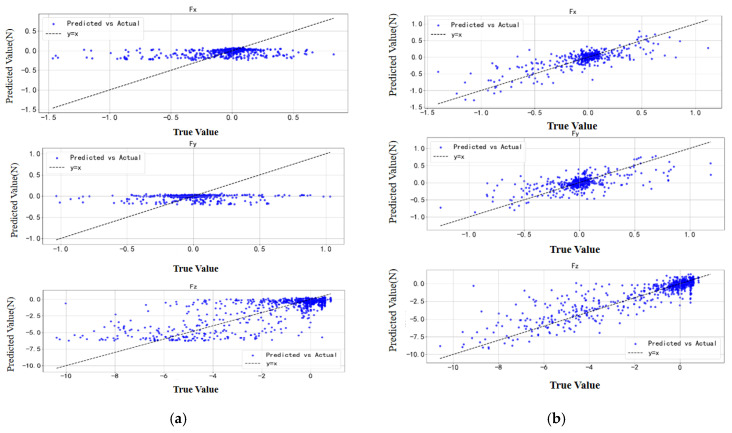
ANN estimation optimization. (**a**) ANN model. (**b**) ANN with multi-feature enhancement.

Similarly, an ablation study was conducted for the ANN-based estimator. As expected, the model achieved the best prediction performance when all features were included, as shown in Table 5. This further confirms that the added feature information positively contributes to the model’s predictive capability. The visualization results are presented in Figure 10. Compared with the GPR model, the ANN model demonstrated superior performance on the test set.

### 4.3. Data Validation

To evaluate the repeatability of the sensing system in detecting contact forces, two sets of experiments were designed based on the aforementioned testing platform. These experiments respectively assess the system’s repeatability under consistent conditions and its robustness during low-frequency random contact scenarios. In the dynamic detection experiments conducted on the test rig, the repeatability of the PVDF sensor was evaluated. By adjusting the height of the lower lifting platform, the contact depth was varied in real time, thereby altering the contact force.

(a) Repeatability Verification

**Figure 11:**
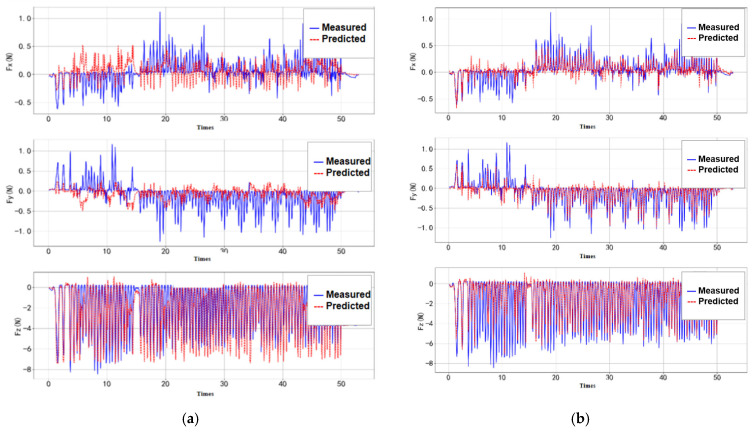
Repeatability verification. (**a**) GPR; (**b**) ANN.

Under repeated contact conditions at 2 Hz, the detection results from both the GPR and ANN models are shown in Figure 11.

o evaluate the performance of the two estimation methods at different frequencies, experiments on three-dimensional contact force detection were conducted at gait frequencies of 1 Hz, 2 Hz, and 5 Hz (see Table 6). In addition to NRMSE, RMSE and R^2^ are introduced to evaluate estimation accuracy. RMSE reflects the average prediction error in Newtons, while R^2^ indicates how well the model captures the variation in true forces. As shown in Table 6, GPR achieves lower RMSE and higher R^2^ across most conditions, especially at 2 Hz. In contrast, ANN shows larger RMSE and lower R^2^, particularly at higher frequencies, indicating reduced reliability under fast gait dynamics. The results show that the GPR model performed consistently across all frequencies, with particularly high accuracy in estimating horizontal forces ($F_{x}$ and $F_{y}$). The ANN model demonstrated an advantage in predicting $F_{z}$ at low frequencies, but its performance degraded significantly at higher frequencies.

(b) Validation under Randomized Gait Frequency Input

Randomized gait frequency input is used to simulate unstructured and highly dynamic contact scenarios that wheel-legged robots may encounter during real-world locomotion. This approach provides a more realistic assessment of the sensing system’s applicability and robustness under complex conditions. The parameter range for randomized step frequency input testing is shown in Table 7.

**Figure 12:**
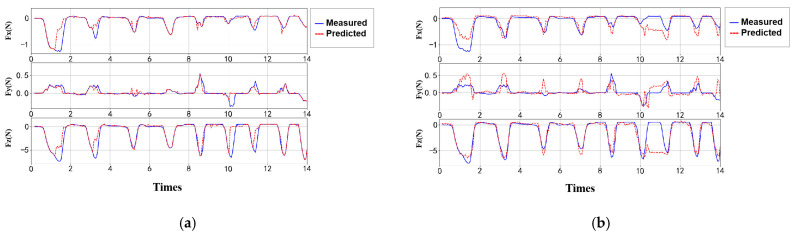
Validation results under randomized gait frequency input. (**a**) GPR validation results. (**b**) ANN validation results.

In the dynamic contact validation, the measured data were estimated using both GPR and ANN models (Figure 12). Both models were able to effectively capture the overall trend of contact force variations. However, differences were observed in the performance along different axes: the GPR model exhibited better fitting accuracy in the x- and y-directions, but showed larger errors in the z-direction, particularly with exaggerated fluctuations under low-frequency conditions. In contrast, the ANN model produced smoother outputs, effectively suppressing noise, and generally delivered higher prediction accuracy and better stability across most scenarios.

**Figure 13:**
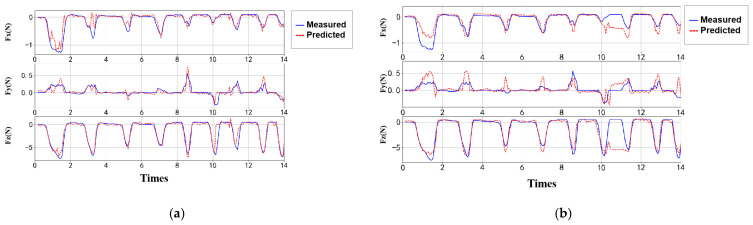
Optimized validation results under randomized gait frequency input. (**a**) GPR model results after temporal cross-validation. (**b**) ANN model results after temporal cross-validation.

Under randomized gait frequency input, the uncontrolled nature of contact and high noise levels led to a noticeable decline in model generalization performance. To enhance robustness, temporal cross-validation was introduced. The performance of the optimized GPR and ANN models on the measured data is shown in Figure 13.

To better reflect real-world applications, three types of data—low-frequency, high-frequency, and gait transition scenarios—were collected for estimation testing. Due to the high dynamic nature and noise interference, the raw data exhibited both abrupt and periodic patterns. To improve model adaptability, temporal cross-validation was introduced, with multiple rolling evaluations performed to ensure output stability across different time windows. In addition to point estimation, the GPR model provides uncertainty intervals, while the ANN model is useful for identifying overfitting tendencies and guiding hyperparameter optimization. As shown in Table 8 and Table 9, the two models demonstrated significant performance differences under varying conditions: the GPR model exhibited more stable performance in predicting Fz, with NRSME ranging from 15.47% to 22.37% and a maximum R2R^2R2 value of 0.7126. The ANN model achieved slightly higher accuracy in low-frequency conditions (with a maximum $R^{2}$ of 0.7424), but its performance was less stable during gait transitions. Overall, the GPR model demonstrated greater robustness in complex dynamic environments.

In the estimation of shear forces ($F_{x}$ and $F_{y}$), both models exhibited relatively large errors. The GPR model achieved NRSME values ranging from 9.11% to 15.56%, with a maximum R^2^ of 0.7116 (for $F_{x}$ under low-frequency conditions). In contrast, the ANN model showed a wider NRSME range (9.93–20.24%) and a lower peak R^2^ of only 0.5745, with its performance dropping significantly during gait transitions, where R2R^2R2 decreased to as low as 0.0179. The ANN model also exhibited higher RMSE values than GPR, indicating greater sensitivity to noise. Overall, both models demonstrated reliable performance in predicting normal forces ($F_{z}$); however, there is still room for improvement in shear force ($F_{x}$, $F_{y}$) estimation, which remains limited by sensor sensitivity, noise interference, and feature extraction strategies.

## 5. Conclusions

This study addresses the challenge of foot–ground contact state perception in wheel-legged robots by proposing a systematic solution that integrates finite element analysis (FEA), global sensitivity analysis (GSA), and machine learning-based estimation. A finite element model of the pneumatic tire was established to reveal the strain distribution characteristics under foot-end loading. Based on the Sobol method for global sensitivity analysis, the optimal placement positions for PVDF piezoelectric film sensors were identified, and the effectiveness of the sensor deployment strategy was validated both theoretically and experimentally. In future work, we plan to introduce a mass–spring–damper (MSD) model to complement the current FEA framework. The MSD model is expected to enhance the accuracy and smoothness of dynamic contact force estimation over time, particularly during locomotion in complex terrains.

In the dynamic testing phase, a customized experimental platform capable of simulating various gait frequencies was developed to collect representative contact datasets. Data preprocessing and feature engineering were applied to improve data quality. Based on these datasets, Gaussian process regression (GPR) and artificial neural network (ANN) models were constructed for predicting three-dimensional contact forces. The results showed that the GPR model offered advantages in uncertainty estimation and demonstrated strong stability and robustness. The ANN model exhibited superior prediction accuracy in low-frequency scenarios, achieving a minimum NRMSE of 8.04% in normal force estimation. Both models showed good generalization performance under temporal cross-validation, meeting the practical requirements for foot-end state estimation in complex terrains.

Overall, this research improves the accuracy and robustness of contact state detection for wheel-legged robots and provides a feasible approach for the design and optimization of smart tire sensing systems. Future work will focus on enhancing shear force prediction accuracy, optimizing multi-sensor fusion strategies, and exploring transfer learning and adaptive mechanisms to improve model generalization for more effective autonomous decision-making and motion control in dynamically complex environments. Additionally, continuous exploration will be carried out to integrate foot-end sensors with multiple other sensors, such as vision cameras, LiDAR, and inertial measurement units (IMUs). By fusing diverse sensing data, we aim to further enhance the adaptability of wheel-legged robots in unstructured terrains, enabling them to navigate rough and unpredictable environments with higher efficiency and stability.

## Figures and Tables

**Table 1:** Tire parameters used in FEA.

Parameters	Value
Material Model	Mooney–Rivlin
IRHD	43
$C_{10}$	0.144 Mpa
$C_{01}$	0.036 Mpa
$D_{1}$ (Bulk Modulus)	0

**Table 2:** Results of Sobol sensitivity analysis.

Sensor Location	Strain in X-Direction	Strain in Y-Direction
P1	1.4719	0.0662
P2	6.1071	9.4910
P3	29.9733	32.1724
P4	6.6748	10.1924
P5	1.5767	0.0662

**Table 3:** Operational range of the dynamic testing scheme.

Parameter	Value
Contact Position	0–30°
Dynamic Contact Force	0–20 N
Repetitive Contact Frequency	0.8–2 Hz
Signal Sampling Frequency	200 Hz
Tire Pressure	0.2 Mpa

**Table 4:** Ablation study results of the GPR model.

Feature Configuration	Fx NRMSE (%)	Fy NRMSE (%)	Fz NRMSE (%)
Raw Sensor Data Only	10.96	1.33	20.42
Without Slope Features	6.74	7.42	17.88
Without Rolling Mean and Variance	8.74	7.91	17.13
All Features Included	7.53	7.39	16.53

**Table 5:** Ablation study results of the ANN model.

Feature Configuration	Fx NRMSE (%)	Fy NRMSE (%)	Fz NRMSE (%)
Raw Sensor Data Only	8.56	8.50	12.46
Without Slope Features	7.35	7.76	9.11
Without Rolling Mean and Variance	7.47	7.75	9.82
All Features Included	6.66	7.47	8.04

**Table 6:** Contact force estimation results at 2 Hz gait frequency on the test platform.

	Estimation Parameters	GPR			ANN		
		NRMSE (%)	RMSE (N)	$R^{2}$	NRMSE (%)	RMSE (N)	$R^{2}$
1 Hz	$F_{x}$	12.71	0.2310	−0.3510	14.47	0.2628	−0.7526
	$F_{y}$	9.17	0.2361	−0.0831	14.09	0.3627	−1.5603
	$F_{z}$	14.22	1.2448	−0.3668	13.58	2.3793	0.5702
2 Hz	$F_{x}$	9.03	0.1569	0.3433	13.71	0.2381	−0.5144
	$F_{y}$	9.11	0.2208	0.4215	12.45	0.3016	−0.0808
	$F_{z}$	19.58	1.6984	0.4275	19.91	1.7272	0.4076
5 Hz	$F_{x}$	11.23	0.1515	0.5152	36.12	0.4873	−4.0197
	$F_{y}$	18.55	0.2433	0.2151	36.05	0.4728	−1.9664
	$F_{z}$	20.89	2.5322	0.4426	17.26	2.0916	0.6197

**Table 7:** Parameter range for low-frequency randomized testing.

**Parameter**	**Value**
Contact Position (°)	0–30
Dynamic Contact Force (N)	0–30 N
Footfall Frequency (Hz)	1~5
Signal Sampling Frequency (Hz)	200 Hz

**Table 8:** Performance of the GPR model before and after temporal cross-validation.

Condition		Before Cross-Validation			After Cross-Validation		
		NRMSE (%)	RMSE (N)	R^2^	NRMSE (%)	RMSE (N)	R^2^
Low Frequency	Fx	10.17	0.1416	0.7047	10.05	0.1399	0.7116
	Fy	12.13	0.1412	0.6194	12.87	0.1497	0.0706
	Fz	17.81	1.4161	0.6194	15.47	1.2305	0.7126
High Frequency	Fx	11.49	0.2266	0.6313	11.25	0.2219	0.6465
	Fy	9.11	0.1181	0.6911	9.77	0.1266	0.6451
	Fz	21.71	1.9763	0.5424	19.84	1.7332	0.6480
Gait Transition	Fx	15.37	0.1710	0.3885	15.56	0.1731	0.3730
	Fy	10.77	0.1921	0.1657	10.36	0.1848	0.2280
	Fz	22.37	2.0179	0.3816	20.12	1.8144	0.5001

**Table 9:** Performance of the ANN model before and after temporal cross-validation.

Condition		Before Cross-Validation			After Cross-Validation		
		NRMSE (%)	RMSE (N)	R^2^	NRMSE (%)	RMSE (N)	R^2^
Low Frequency	Fx	16.25	0.2262	0.2445	16.19	0.2255	0.2496
	Fy	13.09	0.1523	0.0362	11.17	0.1300	0.2977
	Fz	14.64	1.1640	0.7424	18.42	1.4647	0.5922
High Frequency	Fx	12.34	0.2434	0.5745	17.13	0.3378	0.1800
	Fy	17.77	0.2304	0.1756	18.90	0.2451	0.3301
	Fz	21.85	1.9889	0.5362	16.42	1.4948	0.7380
Gait Transition	Fx	20.24	0.2251	0.0629	18.35	0.2041	0.1262
	Fy	9.93	0.1771	0.2883	11.66	0.2081	0.0179
	Fz	20.17	1.8189	0.4965	15.43	1.3914	0.7053

## Data Availability

Data are contained within the article.
